# Evaluating Ethanol-based Sample Preservation to Facilitate Use of DNA Barcoding in Routine Freshwater Biomonitoring Programs Using Benthic Macroinvertebrates

**DOI:** 10.1371/journal.pone.0051273

**Published:** 2013-01-04

**Authors:** Eric D. Stein, Bryan P. White, Raphael D. Mazor, Peter E. Miller, Erik M. Pilgrim

**Affiliations:** 1 Southern California Coastal Water Research Project, Costa Mesa, California, United States of America; 2 Canadian Centre for DNA Barcoding, Biodiversity Institute of Ontario, University of Guelph, Guelph, Canada; 3 United States Environmental Protection Agency, National Exposure Research Laboratory, Cincinnati, Ohio, United States of America; Biodiversity Insitute of Ontario - University of Guelph, Canada

## Abstract

Molecular methods, such as DNA barcoding, have the potential to enhance biomonitoring programs worldwide. Altering routinely used sample preservation methods to protect DNA from degradation may pose a potential impediment to application of DNA barcoding and metagenomics for biomonitoring using benthic macroinvertebrates. Using higher volumes or concentrations of ethanol, requirements for shorter holding times, or the need to include additional filtering may increase cost and logistical constraints to existing biomonitoring programs. To address this issue we evaluated the efficacy of various ethanol-based sample preservation methods at maintaining DNA integrity. We evaluated a series of methods that were minimally modified from typical field protocols in order to identify an approach that can be readily incorporated into existing monitoring programs. Benthic macroinvertebrates were collected from a minimally disturbed stream in southern California, USA and subjected to one of six preservation treatments. Ten individuals from five taxa were selected from each treatment and processed to produce DNA barcodes from the mitochondrial gene cytochrome c oxidase I (COI). On average, we obtained successful COI sequences (i.e. either full or partial barcodes) for between 93–99% of all specimens across all six treatments. As long as samples were initially preserved in 95% ethanol, successful sequencing of COI barcodes was not affected by a low dilution ratio of 2∶1, transfer to 70% ethanol, presence of abundant organic matter, or holding times of up to six months. Barcoding success varied by taxa, with Leptohyphidae (Ephemeroptera) producing the lowest barcode success rate, most likely due to poor PCR primer efficiency. Differential barcoding success rates have the potential to introduce spurious results. However, routine preservation methods can largely be used without adverse effects on DNA integrity.

## Introduction

Molecular methods such as DNA barcoding are emerging as a new tool for species identification [1] and, more recently, environmental biomonitoring [2]–[4]. Biomonitoring (often called bioassessment) is the process of evaluating the overall ecological health of an aquatic environment (such as streams or wetlands) based on the composition and structure of the benthic (bottom dwelling) community. Most often, benthic invertebrate (insect larvae) or instream algal (diatoms or soft-bodied algae) communities are used as the basis of these assessments. Changes in the type or number of organisms, relative to a reference condition, is used to indicate detrimental effects associated with changes in water quality, flow, or physical habitat. Biomonitoring results are commonly used in water quality protection programs as part of ambient monitoring or permit-required compliance assessments.

Routine application of molecular methods to support species identification for biomonitoring will require that each step of sample collection, handling and processing be considered in terms of its potential to affect DNA integrity. Advantages of molecular methods in biomonitoring include the potential to increase the speed, accuracy and resolution of species identification and thereby support effective biomonitoring programs [5]–[9]. Barcoding involves identifying species based on a short DNA sequence from a standardized genetic locus, such as the mitochondrial gene cytochrome c oxidase I (COI) for most metazoans. Using standard molecular methods, DNA is extracted from specimen tissue and sequenced for the 650-bp barcoding region of COI [1]. DNA from unknown specimens collected in benthic samples can be identified by comparing their barcode sequences to a reference library, such as the Barcode of Life Data Systems (BOLD) [10].

The implications of different preservation methods on DNA quality have not been well studied in the context of biomonitoring programs. Traditionally, formalin has been used to preserve marine benthos [11]. Formalin preservation acts by fixing proteins through binding to peptide linkages, which preserves morphological structure but also acts to degrade DNA [12]–[13] Ethanol is the most common preservative for freshwater biomonitoring programs [14], [15]. Initial field preservation is typically done in concentrations ranging from 70–95% at a 2∶1 or 3∶1 volumetric ratio, depending on the program. After dilution from samples and the field matrix, the ultimate concentration is typically around 70% or below. Ethanol is preferred over formalin if specimens will be used for barcoding or other molecular methods because it does not directly affect DNA integrity and, in high enough concentrations (e.g., ≥95%), ethanol denatures proteins that may degrade DNA [16]. Preservation in 95% ethanol at a 5∶1 volumetric ratio is recommended for barcoding studies to ensure preservation and prevention of DNA degradation [17]. However, ethanol is flammable and considered hazardous, which complicates transportation and storage, and creates challenges for use in the field. Furthermore, at high concentrations, ethanol may make tissues friable and difficult to handle for sorting and picking, but the addition of small amounts of glycerin has been suggested as a way to avoid those negative effects. Requiring routine biomonitoring programs to adopt a protocol of 95% ethanol at a 5∶1 ratio would increase cost and logistical challenges and may reduce the incentive for incorporation of molecular methods. Furthermore, the effect on DNA integrity of holding samples for extended periods of time without elutriating out organic debris is poorly understood. Requiring reduced holding times increases sample processing prior to identification and may also impact the ease of incorporating molecular methods into routine programs.

The goal of this study is to evaluate the efficacy of alternative ethanol-based sample preservation methods at maintaining DNA integrity. We evaluated a series of methods that were minimally modified from typical field protocols to identify an approach that can be readily incorporated into existing monitoring programs. Specifically, we evaluated the effect of the following treatments on DNA integrity and the ability to obtain viable barcodes: 1) use of 2∶1 vs. 5∶1 volumetric ratio of 95% ethanol to field sample, 2) preservation in 70% vs. 95% ethanol following initial field preservation, 3) addition of 5% glycerin to reduce tissue friability, and 4) sample holding times at room temperature for up to 6 months. These treatments reflect realistic tradeoffs between optimized preservation of DNA and minimum cost or logistical constraints.

## Methods

### Study sites, sample collection, and preservation treatments

Benthic macroinvertebrates were collected from Bear Creek, a tributary to the West Fork of the San Gabriel River, north of Los Angeles, California, that is minimally disturbed and drains an undeveloped watershed. Past sampling in this area produced a diverse and abundant assemblage of macroinvertebrates that is representative of the taxa typically collected during routine biomonitoring in southern California [18].

Samples were collected from a variety of microhabitats via kick seining using a D-frame net. Samples from pools, riffles, glides, and debris jams were combined to generate a large composite sample, from which ∼0.5 L aliquots were removed and preserved in six different ethanol treatments (Table 1). All samples included abundant organic matter and sediment, and remained non-elutriated and unsorted until processed for DNA extraction. Upon returning to the lab, field samples were drained and replenished with fresh ethanol within 24 hours, and again within 48 hours (for those samples not already sorted). Treatments A–C were sorted, tissues pulled and placed into 96-well plates for DNA extraction within seven days of field collection. Treatments D–F were refrigerated in the dark at 2°C for 30 days (D–E) or 6 months (F) and then sorted, tissue pulled, and plated. Treatment A represents the traditional sampling protocol, and the other treatments represent increasing degrees of modification of this protocol to achieve higher (or more rapid) concentrations of ethanol.

**Table 1:** Preservation treatments showing initial and replenishment concentrations of ethanol, days held in field matrix, day at which DNA extraction was performed, and number of replicates per treatment.

Treatment	Initial preservative	Initial ratio	Replenishment	Days Held in Field Matrix	DNA Extraction	# Replicates
A	95% ethanol	5∶1	95% ethanol	7 d	50 d	3
B	95% ethanol	2∶1	95% ethanol	7 d	50 d	2
C	95% ethanol+5% glycerin	2∶1	95% ethanol+5% glycerin	7 d	50 d	2
D	95% ethanol	2∶1	95% ethanol	30 d	50 d	2
E	95% ethanol	2∶1	70% ethanol	30 d	50 d	2
F	95% ethanol	2∶1	70% ethanol	6 m	174 d	2

After the desired holding time was reached, samples were picked and sorted to Order or Family for barcoding analysis. Two to three replicates were created for each treatment. For each replicate, approximately 10 individuals from each of five taxa were processed for barcoding (Table 2). Taxa were selected a) if they were present in sufficient numbers to be used in all treatments and, b) to represent a range of specimens commonly encountered in routine biomonitoring, and c) to encompass those where past barcoding efforts have produced between moderate and high success rates. Taxa with known poor success rates (e.g., Ostracoda) were not considered.

**Table 2:** Higher taxa (i.e. Class/Order), lower taxon (i.e. Family/Genus), and individuals per replicate.

Higher Taxa	Taxa	Individuals Per Replicate
Ephemeroptera	Leptohyphidae (*Tricorythodes*)	8–10
Trichoptera	Brachycentridae, Lepidostomatidae, Sericostomatidae	7–10
Coleoptera	Elmidae	5–12
Diptera	Chironomidae	10
Gastropoda	Physida/Others	4–10

### DNA extraction, amplification and sequencing

Plates of tissues were shipped to the Candian Centre for DNA Barcoding (CCDB), where the standard COI DNA barcode was sequenced from each specimen using highly automated protocols and a 96-well plate format established at the CCDB by Ivanova et al. [19] and http://www.ccdb.ca/pa/ge/research/protocols. Plates from treatments A–E were held prior to shipment to the CCDB and extracted at the same time. The interval between sample collection and DNA extraction was 50 days for treatments A–E and 174 days for treatment F (Table 1). Once plates were received by CCDB, the well caps were removed to allow the ethanol to completely evaporate. Upon complete evaporation, lysis solution was added to plates, followed the next day by DNA extraction. DNA extracts were PCR amplified using the universal forward and reverse primer-pair C_LepFolF and C_LepFolR respectively (Table 3). If initial amplifications were unsuccessful, DNA extracts underwent additional PCR with primer-pair combinations listed in Table 4. We used both a combination of alternative PCR amplification (i.e. sequencing for 658 bp using alternative primer-pairs) and targeted smaller barcode sizes (∼400 bp). PCR products were bidirectionally sequenced using Sanger sequencing with BigDye v3.1 using an ABI 3730×l DNA Analyzer (Applied Biosystems, Foster City, CA). Sequences and detailed information about all specimens were stored on the Barcode of Life Data Systems [10] and can be accessed there via the project code CFWPA. Sequence data were exported from BOLD and aligned using the MUSCLE algorithm [20] in MEGA 5.05 [21].

**Table 3:** Primers, direction, primer sequence and citation (if available) for all sequences from this study.

Primer name	Direction	Primer Sequence (5′ -> 3′)	Citation
C_GasF1_t1	Forward	TGTAAAACGACGGCCAGTTTTCAACAAACCATAARGATATTGG	CCDB, unpublished
MGasF1_t1	Forward	TGTAAAACGACGGCCAGTATAAGATTTCCTCGWWTRAATAATA	CCDB, unpublished
LCO1490	Forward	GGTCAACAAATCATAAAGATATTGG	[25]
MLepF1	Forward	GCTTTCCCACGAATAAATAATA	[26]
C_LepFolF	Forward	ATTCAACCAATCATAAAGATATTGG	[6]
LepF1	Forward	ATTCAACCAATCATAAAGATATTGG	[6]
dgLCO-1490	Forward	GGTCAACAAATCATAAAGAYATYGG	[27]
LCO1490_t1	Forward	TGTAAAACGACGGCCAGTGGTCAACAAATCATAAAGATATTGG	CCDB, unpublished
GasR1_t1	Reverse	CAGGAAACAGCTATGACACTTCWGGRTGHCCRAARAATCARAA	CCDB, unpublished
MGasR1_t1	Reverse	CAGGAAACAGCTATGACTCCTGTWCCWRCWCCWCCTTC	CCDB, unpublished
HCO2198	Reverse	TAAACTTCAGGGTGACCAAAAAATCA	[25]
C_LepFolR	Reverse	TAAACTTCTGGATGTCCAAAAAATCA	[6]
LepR1	Reverse	TAAACTTCTGGATGTCCAAAAAATCA	[6]
dgHCO-2198	Reverse	TAAACTTCAGGGTGACCAAARAAYCA	[27]
HCO2198_t1	Reverse	CAGGAAACAGCTATGACTAAACTTCAGGGTGACCAAAAAATCA	CCDB, unpublished
MEPTR1_t1	Reverse	CAGGAAACAGCTATGACGGTGGRTATACIGTTCAICC	[2]

**Table 4:** Primer pairs used (forward+reverse) and number of sequence reactions each pair was used.

Forward	Reverse	# Sequence Reactions
C_LepFolF	C_LepFolR	1024
LCO1490_t1	MEPTR1_t1	272
MLepF1	HCO2198_t1	272
LCO1490_t1	HCO2198_t1	194
dgLCO-1490	dgHCO-2198	38
LepF1	LepR1	6
C_GasF1_t1	MGasR1_t1	6
MGasF1_t1	GasR1_t1	6
LCO1490	HCO2198	4

On average, specimens required only a single forward and reverse read in order to obtain a useable contiguous sequence, except for Leptohyphidae, which sometimes required additional PCR attempts followed by two or three forward and reverse reads (four or six reads in total) in order to obtain a consensus sequence.

In order to better understand the reasons for poor Leptohyphid sequencing success, these sequences were analyzed by constructing a neighbor-joining tree of available sequences using the Kimura 2-parameter (K2P) [22] nucleotide model in MEGA 5.05 [21]. COI haplotype clusters were subsequently delimited visually using a 2% genetic distance cutoff [3], [4], although haplotype cluster lineages showed considerably larger genetic distances than 2%. COI haplotype cluster delimitation revealed three clusters of Leptohyphid which we designated *Tricorythodes* sp.1, 2 and 3. These three COI haplotype clusters were then analyzed for differing sequencing success by computing the average sequence length within each COI haplotype cluster and the distribution of those COI haplotype clusters across treatments and replicates.

### Data analysis

Sequence data and specimen meta-data were obtained from the BOLD database. Two levels of sequence data were used for analysis of relative effect of preservation on amplification success. First, partial barcodes (defined for our purposes as sequences less than 500 bp in length) were compared, and second full-barcodes of reference quality (i.e. barcode compliant sequence lengths at least 500 bp) were compared. The proportion of successful specimens was calculated per replicate as the fraction of specimens for which either a partial or full barcode were obtained. Both replicate data and specimen data were imported into the R Statistical Package (R v 2.15.1 http://www.r-project.org/) for analysis. Cumulative frequency graphs were created using the ecdf function in R in order to avoid selection of an arbitrary threshold for comparison. Nested Analysis of Variance (ANOVA) was conducted to compare treatments using the aov function in R. In all cases, p<0.05 was used to indicate statistically significant differences between treatments.

## Results

**Figure 1:**
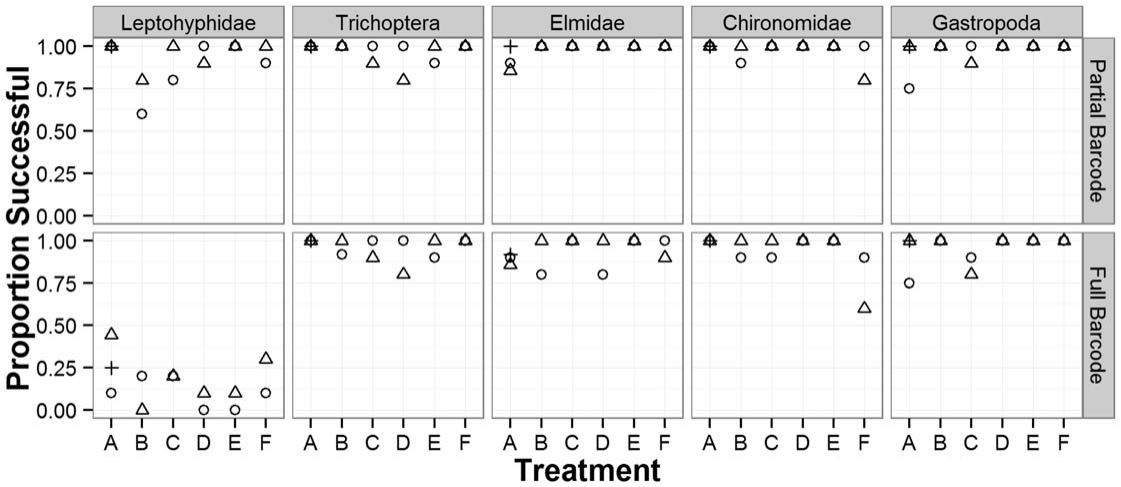
Proportion of specimens successfully amplified for two different sequence length criteria, Partial barcodes (top) and Full barcodes (bottom). Replicates are represented by different shapes (circles, pluses and triangles). Treatment designations (A–F) are as indicated in Table 1.

**Figure 2:**
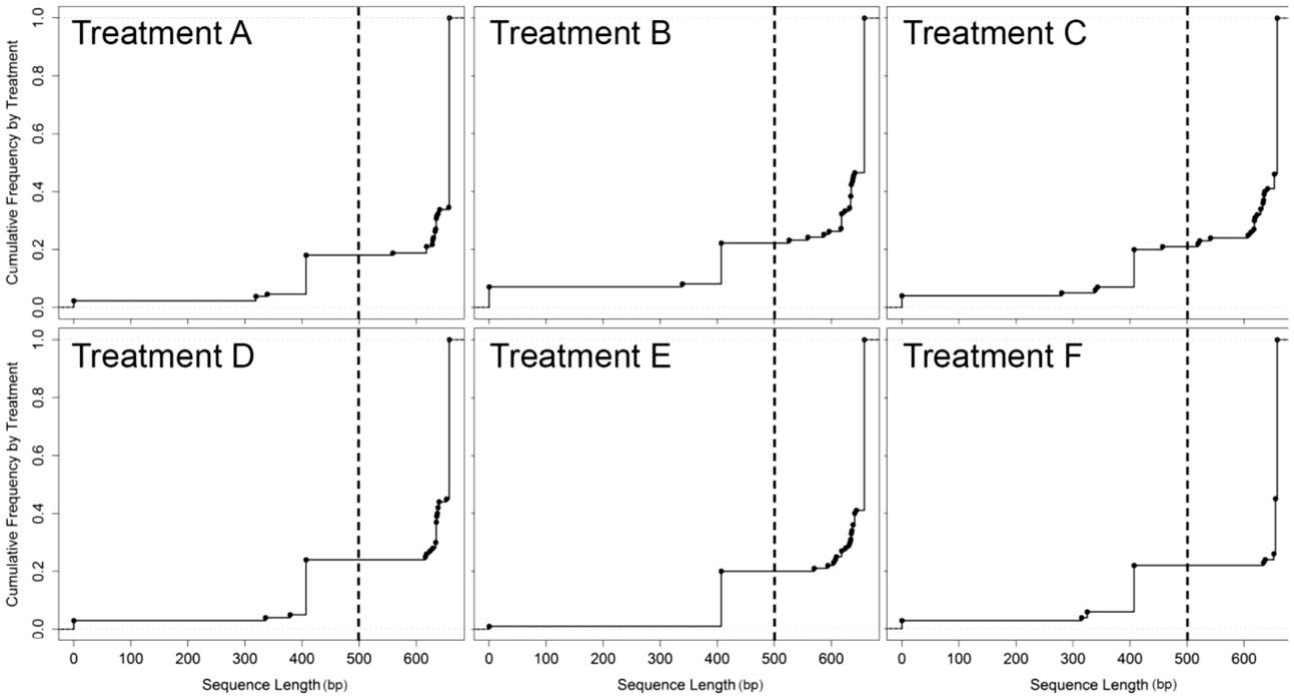
Cumulative frequency graphs of sequence length (base pairs) by treatment. Circles represent the points at which additional sequences are accumulated. Dashed horizontal lines represent the cutoff between partial barcodes (to the left) and full barcodes (to the right).

Sample preservation method (i.e. treatment) had no effect on overall barcoding success rate. We obtained successful COI sequences (i.e. either full or partial barcodes) for between 93–99% of all specimens across all six treatments. For full barcodes, success rates ranged from 68–82% across all taxa with no difference between treatments (Figure 1). There was no significant differences between treatments, replicate nested within treatment, or related interaction according to the nested ANOVA using either the partial barcode or full-barcode criteria (Table 5). To further test the effect of each treatment on barcoding success, we analyzed the frequency of different barcode length. For all treatments, we obtained sequences of 500 base pairs or longer for approximately 70% of specimens (Figure 2).

**Table 5:** Nested ANOVA results for treatment and family effects on amplification success.

Partial Barcode	df	F	p
Rep	1	0.884	0.354
Family (Rep)	4	0.590	0.672
Treatment (Rep)	5	0.450	0.810
Family (Rep)×Treatment (Rep)	20	1.270	0.263
**Full Barcode**			
Rep	1	0.577	0.453
**Family (Rep)**	**4**	**43.4**	**6.73×10^−13^**
Treatment (Rep)	5	0.090	0.993
Family (Rep)×Treatment (Rep)	20	0.874	0.617

Barcode species delimitation analysis and subsequent matching to reference databases revealed that specimens identified to Brachycentridae actually consisted of individuals from the trichopteran families Brachycentridae (109 individuals), Lepidostomatidae (4 individuals), and Sericostomatidae (1 individual). However, PCR success was equally high for all three families, so they were considered one group, Trichoptera, throughout study.

**Figure 3:**
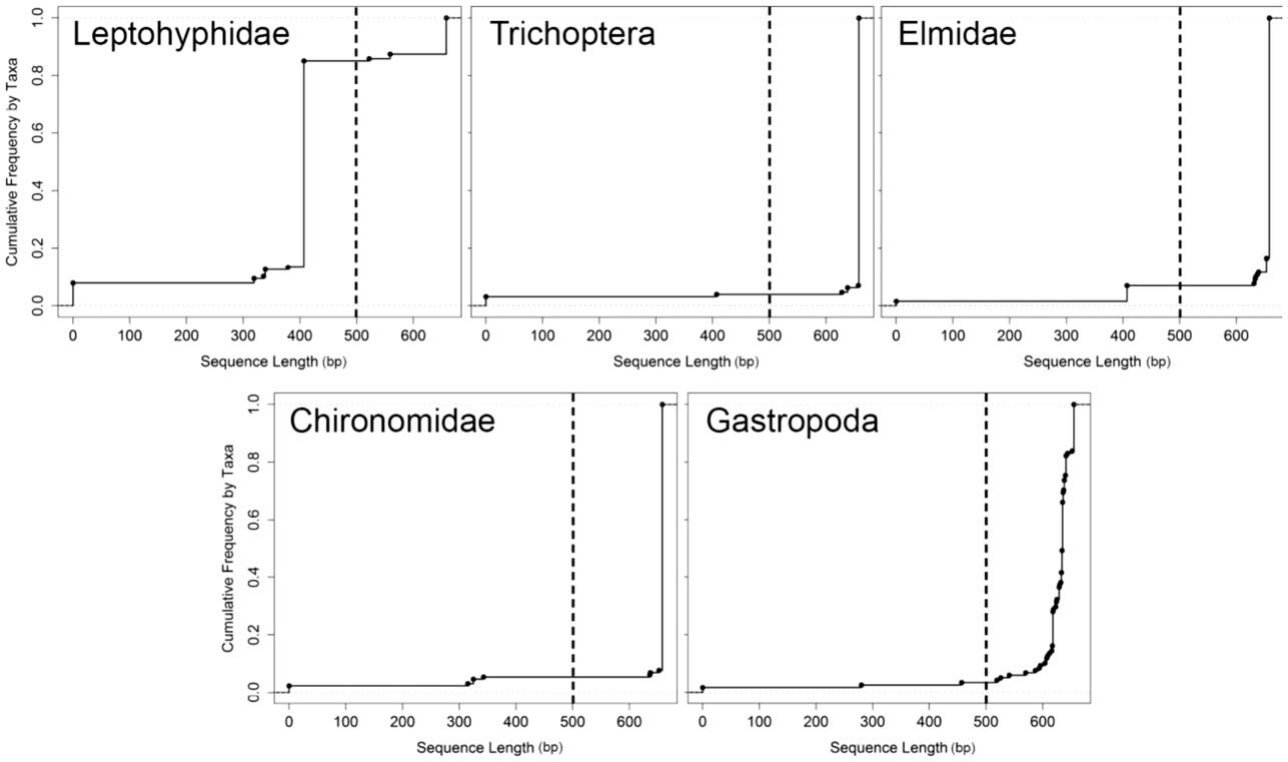
Cumulative frequency graphs of sequence length (base pairs) by lower taxa. Circles represent the points at which additional sequences are accumulated. Dashed horizontal lines represent the cutoff between partial barcodes (to the left) and full barcodes (to the right).

Barcoding success rates varied by taxonomic group for full barcodes, but did not vary for partial barcodes. For full barcodes there was a significant effect of family on the successful generation of full length barcode sequences (*df* = 4, *F* = 43.4, *p*<0.0001). Leptohyphidae (Ephemeroptera) yielded the lowest average success rate with only 18% of specimens successfully amplifying and producing full barcode sequences. Elmidae (Coleoptera) produced the highest average success rate of 93% (Figure 1). There was no significant effect of family on the success of obtaining partial barcode sequences. In terms of the distribution of sequence length, Trichoptera and Gastropoda all produced similar frequency distributions of sequence length whereas Leptohyphidae showed much higher frequencies of barcodes less than 400 base pairs (in the region we refer to as partial barcodes; Figure 3).

Within the problematic leptohyphid specimens, *Tricorythodes* sp. 1 showed the best success with all 9 collected specimens yielding full length barcodes (658 base pairs). *Tricorythodes* sp. 1 was not found at all in treatment B, was found in only 1 replicate in treatments A, D, and E, and was found in all replicates for treatments C and F. *Tricorythodes* sp. 2 was the least abundant species with only 6 collected specimens, yielded the lowest success with an average sequence length of 327 base pairs, and was found in at least one replicate in treatments A, B, C, and D, but none in treatments E and F. *Tricorythodes* sp. 3 was the most abundant of the three COI haplotype clusters with 102 collected specimens with an average sequence length of 423 base pairs and was found in all treatments and replicates. Treatment E, replicate 1 consisted entirely of *Tricorythodes* sp. 3.

## Discussion

Sample preservation methods typically used in routine bioassessment do not appear to adversely affect the ability to obtain successful barcodes for most benthic macroinvertebrates. As long as samples were initially preserved in 95% ethanol, successful sequencing of COI barcodes was not affected by a low dilution ratio of 2∶1, transfer to 70% ethanol, presence of abundant organic matter, or holding times of up to six months. This success is likely due to the fact that the initial preservation in 95% ethanol was sufficient to replace most water in the specimen with ethanol, thereby arresting potential DNA degradation. Such rapid desiccation of benthic macroinvertebrates following ethanol preservation has been previously documented [23]. Successful barcodes were even obtained for the elmid beetles which had the highest success rate for generating full barcodes, even though they generally are considered difficult to preserve due to their heavily sclerotized exoskeleton.

Our results are in apparent contradiction to Baird et al. [24] who noted poor sequencing success when using 70% ethanol preservation of benthic invertebrates and consequently recommended preservation in 95% ethanol at −10°C to limit DNA degradation. However, the source material in the Baird et al. study was reference material collected as part of the Canadian Aquatic Biomonitoring Network (CABIN). The specimens ranged from 1–23 years old and were originally fixed in 10% formalin before being transferred to 70% ethanol for long term storage. Therefore, Baird et al.'s findings apply more to the use of formalin than to initial preservation in ethanol, which is how we recommend samples be handled when molecular methods such as DNA barcoding are integrated into biomonitoring programs.

Pilgrim et al. [4] suggested that sample preservation methods may be a contributing factor to lower than desirable rates of successful barcoding. We found this to not be the case, rather primer limitations were a larger source of failure than preservation methods in this study. For example, we achieved low success rates with barcoding the Leptohyphidae due to difficulty with DNA amplification, not due to sample preservation. Different taxa had different success rates showing that PCR amplification problems outweighed any differences due to sample preservation. Differential effectiveness of primers at amplifying different taxonomic groups can lead to apparent treatment effects if the composition of specific samples varies. For example, if a sample is dominated by taxonomic groups that typically experience low barcode success, it may lead to erroneous differences between treatments. Primer biases could be partially addressed by developing family level partial barcode primers for difficult groups (i.e., Leptohyphidae) as full-barcodes for many of those difficult groups are readily available. The benefit of partial barcodes is illustrated by the increased success we observed when using partial barcodes as opposed to full COI barcodes. Alternately, obtaining a larger piece of mitochondrial sequence containing the 5′ region of COI across taxa could aid the development of full-length, family specific barcode region primers, and help to eliminate the possibility of taxa-specific DNA degradation effects.

This study provides promise for the ability to employ existing standard sample preservation methods with 95% ethanol for freshwater bioassessment when including DNA barcoding. In California, most routine biomonitoring programs already use an initial preservation in 95% ethanol (at a 2∶1 or 3∶1 ratio), with the goal of achieving a final concentration of 70% ethanol in the field. For those programs that currently use 70–80% ethanol for the initial field preservation, only a minor adjustment to 95% for the initial preservation with subsequent ethanol replacement in the lab is necessary to ensure DNA integrity. This adjustment represents a minor additional cost relative to the benefits obtained by having samples available for barcoding or other molecular methods. Maintaining existing protocols, which are often institutionalized through standard operating procedures or regulatory requirements, eliminates a potential impediment to incorporating molecular methods into ongoing biomonitoring programs. For those that have concerns about tissue friability of some specimens, the addition of glycerin may reduce this problem and it poses no difficulties for DNA-based work. The results of this work suggest that agencies that currently favor formalin preservation due to concerns about requirements of large ethanol volumes can develop collection protocols using ethanol preservation that are not needlessly onerous.
